# Growth, Cannibalism, and 5-TH Metabolism in Pufferfish (*Takifugu obscurus* ♀ × *Takifugu rubripes*): The Role of Graded Levels of Dietary Tryptophan

**DOI:** 10.1155/2023/6693175

**Published:** 2023-09-09

**Authors:** Yuliang Wei, Xishuai Cui, Zhibing Zhou, Qiang Ma, Houguo Xu, Mengqing Liang

**Affiliations:** ^1^Yellow Sea Fisheries Research Institute, Chinese Academy of Fishery Sciences, 106 Nanjing Road, Qingdao 266071, China; ^2^Laboratory for Marine Fisheries Science and Food Production Processes, Laoshan Laboratory, 1 Wenhai Road, Shandong, Qingdao 266237, China

## Abstract

The objective of this study was to investigate the potential effect of graded levels of tryptophan on the growth, cannibalism, and 5-hydroxytryptpamine (5-TH) metabolism in pufferfish (*Takifugu obscurus* ♀ × *Takifugu rubripes* ♂). A 63-day feeding trial was performed wherein pufferfish were fed four diets. Three experimental diets were formulated with various levels of tryptophan based on the control diet. Four diets were named as T1, T2, T3, and T4, corresponding to 4.30, 7.80, 14.90, and 23.70 g kg^−1^ tryptophan of dry diet. Final body weight, weight gain, and specific growth rate were similar between the T1 and T4 groups, but exhibited a significantly increased trend compared to the T2 group. Although survival rate was not affected by various levels of dietary tryptophan, intraspecific cannibalism was significantly reduced in the group fed with highest level of tryptophan (T4). For free amino acid in brain, the concentration of tryptophan was the highest in the T3 group and the lowest in the T2 group, while phenylalanine, tyrosine, and methionine showed an opposite trend between those two groups. The levels of dietary tryptophan not only affected the expression of aromatic amino acid transporter TAT1, but also affected the expression of B0AT1, B0AT2, and 4F2hc in intestine, as well as B0AT1, y^+^LAT1, and LAT2 in brain. The activity of tryptophan hydroxylase (TPH) in serum increased with the increase of dietary tryptophan, and the expression of TPH1 in brain upregulated in the excessive tryptophan groups (T2, T3, and T4). MAO activity in serum as well as its gene expression in brain and intestine showed a decreased trend in the T4 group. In conclusion, excessive tryptophan (23.70 g kg^−1^ of dry diet, corresponding to 50.3 g kg^−1^ of dietary protein) in feed could mitigate cannibalistic behavior of pufferfish and promote the growth, and the reason for this effect might affect the metabolism of 5-TH *in vivo*.

## 1. Introduction

Tryptophan is an essential amino acid for fish which participate in the body proteins synthesis. Available data indicated that tryptophan requirements for fish ranged from 0.3 to 1.3% of dietary protein levels [1]. Meanwhile, tryptophan, as a functional amino acid, was found to improve animal welfare, including mitigating crowding and netting stress in meager (*Argyrosomus regius*) [2], the stress due to tank color in rainbow trout (*Oncorhynchus mykiss*) [3], thermal stress in Atlantic cod (*Gadus morhua*) [4], and Indian major carps (*Labeo rohita*) [5], the stress of constant aeration in Persian sturgeon (*Acipenser persicus*) [6]. Tryptophan was a precursor for the synthesis of the neurotransmitter serotonin (5-hydroxytryptpamine, 5-TH), which plays an important role in stress mitigation [7]. In addition, some previous studies reported that tryptophan had a suppressive effect on aggressive behavior and cannibalism of some fish species through the 5-HT pathway [8–13]. Reducing intraspecific aggression and cannibalism was very important in fish rearing because it was associated with survival rate [14–16].

Pufferfish are one of cultured fish species with a very high market price in China. In 2021, according to China Fishery Statistical Yearbook (2022), the production of pufferfish was up to 29,940 tons. In particular, obscure pufferfish (*Takifugu obscurus*) and tiger puffer (*Takifugu rubripes*) had become the most important cultured species, because they were the only two pufferfish that the Chinese government conditionally allows to be processed and operated [17, 18]. However, obscure pufferfish and tiger puffer had sharp teeth and often cannibalize each other, which were considered as a typical cannibalistic species [19]. For instance, Hu et al. [20] reported that the mortality and injury rates (the fin damage) of pufferfish were 6.7% and over 20%, respectively, even if feeding ration was set at 3% of fish body mass, whereas they would reach 25% and over 50%, respectively, when feeding ration was only 1%. Due to mass mortality caused by cannibalism, it had become an important issue in the pufferfish farming [21]. Some studies had focused on the effects of rearing conditions such as stocking density on the cannibalism of pufferfish [22, 23]. Apart from that, tryptophan had been found to play an important role in mitigating cannibalism of grouper (*Epinephelus coioides*) [15], pikeperch (*Sander lucioperca*, L.) [8], and Pabda (*Ompok bimaculatus*, Bloch) [14]. For this reason, tryptophan-enriched feed should need to be studied in pufferfish as a nutritional tool for cannibalism.

Thus, the objective of this study was to investigate the potential effects of dietary tryptophan supplementation on the growth, cannibalism, and 5-TH metabolism in pufferfish.

## 2. Materials and Methods

### 2.1. Experimental Diets

The basal diet (T1) contained fish meal, brewer's yeast, corn protein concentrate, and gelatin as the main protein source. Graded levels of crystalline tryptophan (3, 9, and 18 g kg^−1^) were supplemented to the basal diet, at the expenses of glycine and glutamic acid (1:1). The supplemental diets were considered as experimental diets and would be referred as T2 (3 g kg^−1^ crystalline tryptophan), T3 (9 g kg^−1^ crystalline tryptophan), and T4 (18 g kg^−1^ crystalline tryptophan). The formulation and proximate composition of all diets are given in Table 1. Proximate analysis of the diets showed about 480 g kg^−1^ crude protein, about 78 g kg^−1^ crude lipid, and about 60 g kg^−1^ ash. Amino acid composition of the diets is given in Table 2. Amino acid analysis showed that concentrations of tryptophan in the T1, T2, T3, and T4 diets were 4.30, 7.80, 14.90, and 23.70 g kg^−1^ of dry diet, respectively.

### 2.2. Experimental Fish and Feeding Trail

Feeding trial was conducted at Jiangsu Zhongyang Group Co. Ltd. (Haian, Jiangsu, China). The hybrid of *Takifugu obscurus* (♀) × *Takifugu rubripes* (♂) were transported to an experimental culture system, and then given a commercial diet for 1 week to adapt to the experimental conditions. A total of 480 fish (an average body weight of 38 g) were randomly distributed into 12 fiberglass tanks (diameter, 150 cm; height, 60 cm) with 40 fish per tank. Each experimental diet was assigned to triplicate tanks. Fish were fed by hand three times a day (06:30, 12:00, and 17:00) to apparent visual satiation (over 80% of fish per tank had no obvious feeding behavior). The growth trial lasted for 63 days. In the experimental system, 1/3^rd^ water was exchanged daily using borewell water and continuous aeration was ensured in each tank throughout the experimental period. In all the experimental tanks, the water quality parameters were observed to be within the tolerable levels (water temperature was 25–28°C, the level of dissolved oxygen was above 7 mg L^−1^, and nitrogen compounds were 0.4–0.8 mg L^−1^).

### 2.3. Sample Collection

At the end of the feeding trial, all fish were deprived of feed for 24 hr, and then were bulk-weighted to calculate growth parameters. Before the sampling, fish were anesthetized with tricaine methanesulfonate (MS-222, 30 mg L^−1^). Fish from each tank were individually checked for bite marks and counted to calculate the rate of damage. Then, six fish per tank were collected to take blood samples from the caudal vein by using heparinized needles. Serum was obtained by centrifuging at 3,000 *g*, and stored at −80°C for further analysis. After blood sampling, fish were euthanized with lethal dose of MS-222 (200 mg L^−1^) and dissected to collect brain and intestine. The tissue samples were also stored at −80°C for further analysis. In the current experiment, all procedures were performed with the permission of Institutional Animal Care and Use Committee of the Yellow Sea Fisheries Research Institute (Certificate number: 352021069).

### 2.4. Growth Parameters and the Rate of Damage Calculations

The growth parameters and the rate of damage were calculated from the following formulas:

Weight gain=100 × (final weight − initial weight)/initial weight, 

Specific growth rate=100 × [ln(final weight) − ln(initial weight)]/feeding days, 

Feed intake=100 × total feed intake/[feeding days × (final weight+initial weight)/2], 

Feed efficiency ratio=body weight gain/dry feed intake, 

Protein efficiency ratio=(final weight − initial weight)/protein intake, 

Protein productive value=(final protein content − initial protein content)/protein intake, 

Survival rate=100 × (final fish number/initial fish number), 

The rate of damage=100 × (the number of injured fish)/(the number of remaining fish).

### 2.5. Analytical Methods

#### 2.5.1. Chemical Analysis

Proximate composition of experimental diets and whole body samples were analyzed as follow: dry matter was determined by drying the samples at 105°C until getting a constant weight; crude protein was determined by the Kjeldahl method (*N* × 6.25) (Foss, 2300, Sweden); crude lipid was determined by petroleum ether extraction (Foss, 2050, Sweden); ash was determined by combustion at 550°C for 16 hr. Experimental diets were hydrolyzed using 6 mol L^−1^ HCl for the determination of total amino acids and using 6 mol L^−1^ NaOH for the determination of tryptophan. Lyophilized brain samples were deproteinized by trichloroacetic acid (6%) to determine free amino acids. Concentrations of all amino acids were measured by the amino acid analyzer (Hitachi, L-8900, Japan).

#### 2.5.2. The Analysis of 5-Hydroxytryptamine, 5-Hydroxyindole Acetic Acid, Tryptophan Hydroxylase, and Monoamine Oxidase Levels

The levels of 5-TH, 5-hydroxyindole acetic acid (5-HIAA), tryptophan hydroxylase (TPH), and monoamine oxidase (MAO) in serum were measured using enzyme-linked immunosorbent assay (ELISA). All commercial ELISA kits (5-TH: Cat. No. ml064304; 5-HIAA: Cat. No. ml901511; TPH: Cat. No. ml541280; MAO: Cat. No. ml466152) were provided by Shanghai Enzyme-Linked Biotechnology (Shanghai, China). The method of ELISA was performed according to the manufacturer's protocol. Experimental procedure had been described in our previous study [24]. Each sample was analyzed in duplicate.

#### 2.5.3. Gene Expression Analysis

Gene expression analysis were conducted as our previous studies [17]. In briefl, total RNA were isolated from intestine and brain by using RNAiso Plus kit (Takara, Japan). The integrity and purity of RNA were evaluated based on the bands of ribosomal RNA (18S:28S) and A260/A280 ratio (1.8–2.0). After the concentration of total RNA was determined, the RNA was reverse-transcribed to cDNA according to the protocol of M-MLV RT Mix kit with gDNA Clean (Accurate Biotechnology, China). Quantitative real time–polymerase chain reaction (qRT–PCR) was carried out in a Roche LightCycler 96 system (Roche, Switzerland) using SYBR Green Premix Pro Taq HS qPCR Kit (Accurate Biotechnology, China) following the recommended protocol. The qRT-PCR was performed in duplicate of each sample to obtain the threshold cycle (Ct) values. The relative gene expression was calculated using the method of 2^−*ΔΔCt*^ [25]. Primer pairs for housekeeping gene and target gene are listed in Table 3. Gene expression was normalized relative to the geometric mean of the Ct values from *β*-actin and RNA polymerase II subunit D (RPSD).

### 2.6. Statistical Analysis

Data were checked for normal distribution using Shapiro–Wilk test and for homogeneity of variances using Levene's test. One-way analysis of variance (ANOVA) followed by Tukey's HSD multiple range test was used to assess significant differences between treatments. The level of significance was obtained when the *P* value was set to less than 0.05. All data were presented as means ± standard error and analyzed using SPSS 16.0 (SPSS Company).

## 3. Results

### 3.1. Growth Performance

Final body weight, weight gain, and specific growth rate in fish fed diets containing 4.30 (T1) and 23.70 (T4) g kg^−1^ tryptophan exhibited a significantly increased trend compared to fish fed diets containing 7.80 (T2) g kg^−1^ tryptophan (Table 4). In addition, although there was no significant difference in survival rate between treatments, the rate of damage due to intraspecific cannibalism was significantly decreased in fish fed an excess of tryptophan (T4). No differences were found in feed intake, feed efficiency ratio, protein efficiency ratio, and protein productive value among the experimental groups.

### 3.2. Whole-Body Proximate Composition and Physical Indicators

Crude lipid and moisture in the whole-body were not affected by different levels of dietary tryptophan (Table 5). However, crude protein and ash in the whole-body were higher in the T2 group relatively to the T3 group. Hepatosomatic index, viscerasomatic index, and condition factor were not affected by dietary treatments.

### 3.3. Free Amino Acid of Brain

Of all essential amino acids, free methionine, phenylalanine, tryptophan, and histidine concentrations in brain were significantly affected by dietary treatments (Table 6). The concentration of tryptophan was the highest in the T3 group and the lowest in the T2 group, while methionine and phenylalanine showed an opposite trend between those two groups. Free histidine concentration increased with the increase of dietary tryptophan, and the highest level was observed in the T4 group. As for nonessential amino acids, only free cystine and tyrosine concentrations in brain showed significant differences among all the groups. Similar to methionine and phenylalanine, tyrosine concentration was the highest in the T2 group and the lowest in the T3 group. Level of cystine exhibited a significant reduction in the T1 group relative to the T2, T3, and T4 groups.

### 3.4. The 5-HT, 5-HIAA, and 5-HIAA/5-HT Ratio in Serum

According to Table 7, no significant differences were observed in serum 5-HT, 5-HIAA, and 5-HIAA/5-HT ratio among all the groups.

### 3.5. Gene Expression of Amino Acid Transporters in Intestine and Brain

**Figure 1:**
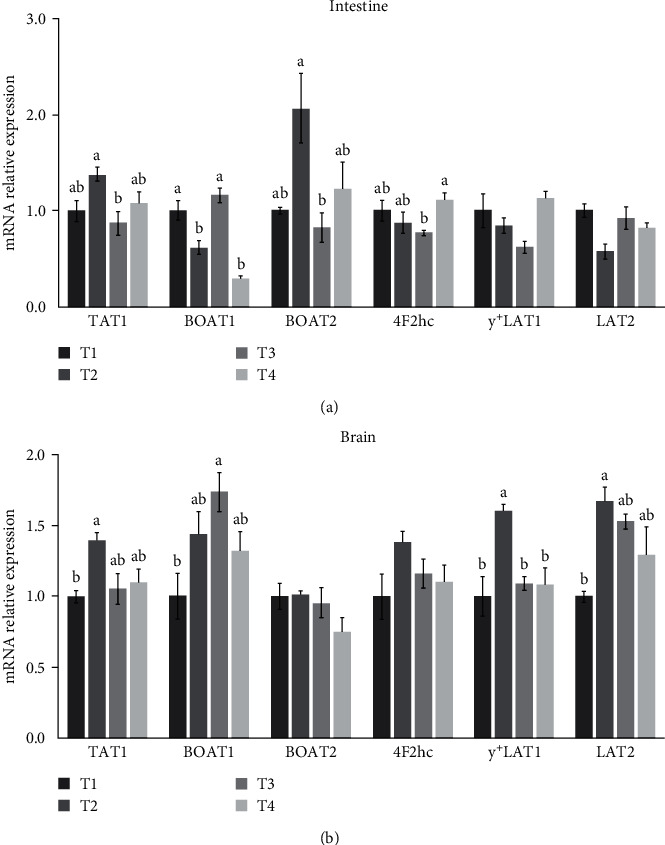
Effects of dietary tryptophan on mRNA expressions of amino acid transporters in intestine and brain (a, b). TAT1: T-type amino acid transporter (SLC16A10); B0AT1: B0 neutral amino acid transporter 1 (SLC6A19); B0AT2:SLC6A15; 4F2hc: 4F2 cell-surface antigen heavy chain (SLC3A2); y^+^LAT1: y^+^l-type amino acid transporter 1 (SLC7A6); LAT2: large neutral amino acids transporter small subunit 2 (SLC7A8). Data are presented as mean ± SEM (*n* = 3). Bars with different letters are not significantly different by Tukey's multiple comparison test (*P* < 0.05).

In intestine, the expressions of TAT1 and B0AT2 were similar among all the groups, with the highest expression in the T2 group, which was significantly higher than that in the T3 group (Figure 1(a)). The lowest expression of 4F2hc in intestine was observed in fish fed the T3 diet. B0AT1 mRNA level of intestine in the T1 and T3 group was significantly higher than that in the T2 and T4 group.

Regarding the expressions of amino acid transporters in brain, Figure 1(b) showed that the expressions of TAT1, y^+^LAT1, and LAT2 were significantly higher in fish fed the T2 diet than that fish fed the T1 diet. B0AT1 mRNA level in brain was significantly affected by the dietary tryptophan level, which fish fed the T3 diet had higher expression than that in fish fed the T1 diet.

### 3.6. Activities of Enzymes and Gene Expression Related to Serotonergic Pathway

**Figure 2:**
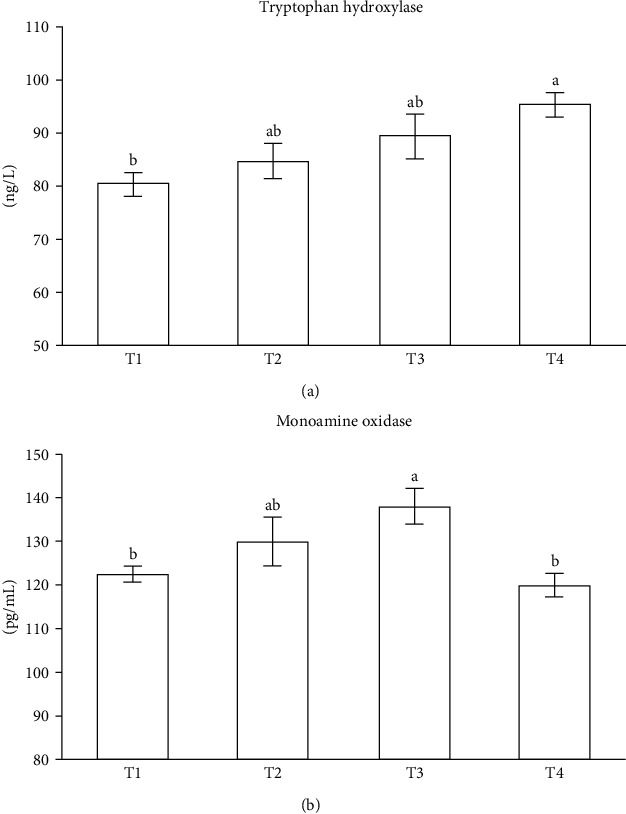
Activities of enzymes related to serotonergic pathway (TPH and MAO) in serum (a, b). TPH: tryptophan hydroxylase; MAO: monoamine oxidase. Data are presented as mean ± SEM (*n* = 3). Bars with different letters are not significantly different by Tukey's multiple comparison test (*P* < 0.05).

Activities of enzymes related to serotonergic pathway (TPH and MAO) in serum are presented in Figure 2. The activity of TPH increased with the increase of dietary tryptophan. For MAO, the highest activity was observed in the T3 group, which was significantly higher than that in the T1 and T4 group.

**Figure 3:**
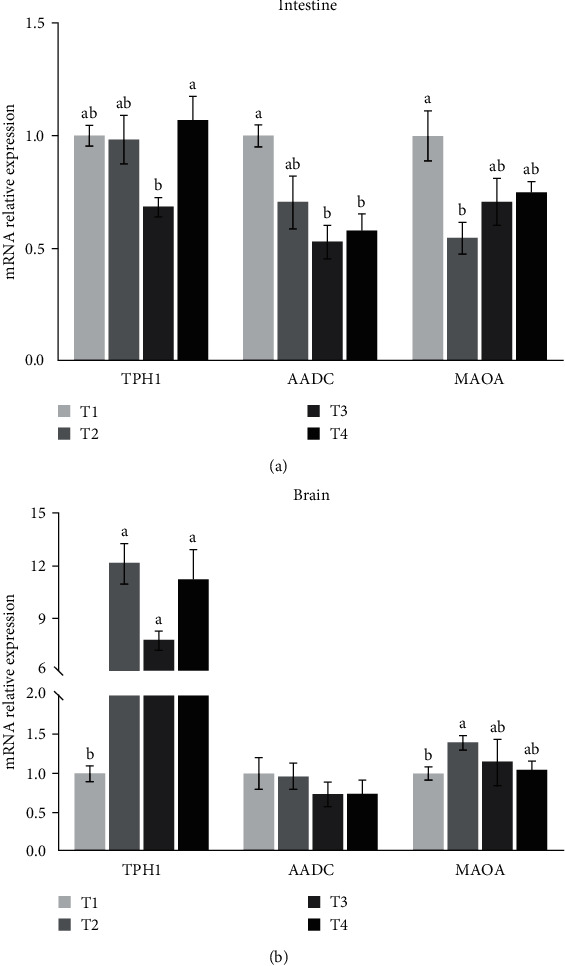
Effects of dietary tryptophan on mRNA expressions of TPH1, DDC, and MAO in intestine and brain (a, b). TPH1: tryptophan hydroxylase 1; DDC: dopa decarboxylase; MAO: monoamine oxidase. Data are presented as mean ± SEM (*n* = 3). Bars with different letters are not significantly different by Tukey's multiple comparison test (*P* < 0.05).

The effect of dietary tryptophan levels on the expression of TPH1, DDC, and MAO in intestine was different from that in brain (Figure 3). The expression of TPH1 in intestine was the highest in the T4 group, which was significantly higher than that in the T3 group. In brain, the level of TPH1 expression in the T2, T3, and T4 groups was significantly higher than that in the T1 group. The expression of DDC in intestine of the T1 group was the highest and significantly higher than in the T3 and T4 groups, but there was no significant difference in brain between treatments. The expression of MAO in intestine was the highest in the T1 group, but the highest in brain was in the T2 group.

## 4. Discussion

As an essential amino acid, to our knowledge, the requirement of tryptophan for obscure pufferfish and tiger puffer had yet to be established. However, according to the review of Hoseini et al. [1], dietary tryptophan requirement for different fish species ranged from 3 to 13 g kg^−1^ of dietary protein level. Thus, dietary tryptophan levels in this study were designed from 4.3 to 23.7 g kg^−1^ of dry diet, corresponding to 9.0–50.3 g kg^−1^ of dietary protein. The growth exhibited the lowest point at 7.8 g kg^−1^ tryptophan of dry diet (16.5 g kg^−1^ of dietary protein), and then gradually increased as the increase of dietary tryptophan level. Final body weight, weight gain, and specific growth rate in fish fed 23.7 g kg^−1^ tryptophan of dry diet (50.3 g kg^−1^ of dietary protein) were close to those in fish fed 4.3 g kg^−1^ tryptophan of dry diet (9.0 g kg^−1^ of dietary protein). Contradictory results of the effect of optimum dietary tryptophan levels on maximum growth had been reported in some fish species. For example, tryptophan requirement for *Labeo rohita* was ranged from 9.0 to 11.3 g kg^−1^ of dietary protein [26, 27], while increasing tryptophan levels to 30.5–51.7 g kg^−1^ under ammonia nitrogen, temperature, or salinity stress can further improve growth [28, 29]. Similarly, in *Cirrhinus mrigala*, it was also found that although optimum dietary tryptophan level was estimated to be 9.5–12.0 g kg^−1^ of dietary protein [30, 31], the addition of 13.6–27.2 g kg^−1^ of dry diet to the based diet would further promote growth under crowding stress [32]. Thus, when some fish were exposed to stress conditions, more tryptophan supplies in feed may need to be ready for optimum growth of the fish. One possible explanation was that tryptophan was a functional amino acid, which that was not only used for protein synthesis but also involved in 5-HT synthesis [1]. In this study, since pufferfish are the fish with cannibalistic or aggressive behavior (this cannibalistic behavior can be predicted based on fish with bite marks at head region or tail region), it was possible that an excess of tryptophan in feed would mitigate intraspecific cannibalism. And then, mitigated cannibalism may lead to promote growth of pufferfish due to reduced competition for feed when a certain amount of tryptophan was supplemented into the feed [33]. It was supported by the data from the rate of damage (fish with bite marks were recorded after feeding trail), where the lowest level was observed in fish fed 23.7 g kg^−1^ tryptophan of dry diet.

Studies on the effect of tryptophan on the stress of fish, including cannibalism and aggression, mainly focus on its participation in 5-HT metabolism as a precursor [8, 13, 34]. TAT1 was currently the only known transporter for aromatic amino acids [35–37]. Like phenylalanine and tyrosine, tryptophan is an aromatic amino acid. Meanwhile, given that 5-HT was mainly synthesized in the intestine and brain [33], the expression of TAT1 was analyzed to evaluate the transport and absorption of tryptophan in the intestine and brain. In this study, the expression of TAT1 was upregulated in the intestine and brain of fish fed 7.8 g kg^−1^ of dietary tryptophan. It indicated that dietary tryptophan moderately exceeding the requirements for pufferfish may increase TAT1 expression. However, the brain contained the lowest level of free tryptophan and the highest level of tyrosine or phenylalanine in the 7.8 g kg^−1^ tryptophan group. Therefore, it was speculated that the upregulation of TAT1 expression may mainly promote the transport of phenylalanine and tyrosine. In addition, the expressions of other amino acid transporters (B0AT1, B0AT2, 4F2hc, y^+^LAT1, and LAT2) were determined in the intestine and brain. B0AT1 can transport all the neutral amino acids [38, 39], while B0AT2 mainly transports branched chain amino acids, methionine, and proline [40]. y^+^LAT1 and LAT2 belong to solute carrier (SLC) seven families, which are nonglycosylated light chain transporters. They can form heteromeric amino acid transporters (HATs) with glycosylated heavy chain transporters from the SLC3 family (4F2hc) [41]. The heavy chain is ancillary protein and responsible for the stability and localization of the transporter, whereas the light chain facilitates amino acid transport [42, 43]. 4F2hc–LAT2 can transport small and large neutral l-amino acids, such as cysteine, methionine, phenylalanine, tyrosine and histidine [44]. 4F2hc–LAT2 was evidenced as obligatory exchangers for the aromatic amino acid diffusion by TAT1 transporter [45]. 4F2hc–y^+^LAT1, another one HAT, is also an obligatory exchanger of cationic amino acids and neutral amino acids [42]. The expressions of B0AT1, B0AT2, and 4F2hc in intestine and the expressions of B0AT1, y^+^LAT1, and LAT2 in brain were affected by the levels of tryptophan in feed. It explained why, in addition to tryptophan, dietary tryptophan levels significantly affected the levels of free methionine, phenylalanine, histidine, cystine, and tyrosine in brain. Furthermore, phenylalanine, tyrosine, and methionine are large neutral amino acids (LNAAs) as tryptophan, and their levels in the T2 and T3 groups showed opposite trends to that of tryptophan. It was possible that LNAAs shared the same transporter located at the blood–brain barrier for uptake into the brain, resulting in competitive effects of these amino acids [46, 47].

In the biosynthetic pathway of 5-HT from tryptophan, there are two enzymes involved, namely TPH and aromatic amino acid decarboxylase (AADC). TPH is the rate limiting enzyme that catalyzes the hydroxylation of tryptophan to 5-hydroxytryptophan (5-HTP), whereas aromatic amino acid decarboxylase, catalyzing the decarboxylation of 5-HTP to 5-HT, is present in all cells of the body and have high activity in that metabolic pathway [48]. In this study, the activity of TPH increased gradually with the increase of dietary tryptophan. This result was supported by the expression of TPH1 in brain, which was significantly upregulated when an excess of tryptophan was supplemented to the diets. The effect of dietary tryptophan on TPH in this study was similar to that in piglets [49] and broilers [50]. It indicated that the synthesis of 5-HT in pufferfish might be enhanced by supplementing tryptophan into diets. In addition, the expression of TPH1 in intestine was not increased with the increase of dietary tryptophan, and even significantly decreased in the T3 group. The possible reason was that some tissues of pufferfish needed to control the production of 5-HT by downregulating TPH1 expression after an excess of tryptophan provided [50]. Meanwhile, since dopa decarboxylase (DDC) was responsible for encoding the AADC enzyme [51], this result was also supported by the expression of DDC in intestine, where its expression was downregulated with the increase of dietary tryptophan. Höglund et al. [33] proposed that the release of 5-HT, the concentration of the catabolite 5-hydroxyindole acetic acid (5-HIAA) (the main 5-HT metabolite) and the 5-HIAA/5-HT ratio could be used to reflect the rate of 5-HT synthesis. In zebrafish (*Danio rerio*), Teixeira et al. [52] reported that the level of 5-HT in brain showed a linear increase with the increasing level of dietary tryptophan. In rainbow trout (*Oncorhynchus mykiss*), Lepage et al. [9] and Winberg et al. [11] found that dietary tryptophan did not affect the level of 5-HT, but elevated the levels of 5-HIAA or the 5-HIAA/5-HT ratio in the telencephalon and hypothalamus, rather than in the brain stem and optic tectum. In our study, due to the small size of the fish, the amount of brain tissue sampled was very limited, only enough for the analysis of free amino acids and gene expression, thus the levels of 5-HT and 5-HIAA were measured in serum. The present results showed that 5-HT, 5-HIAA, and the ratio of 5-HIAA/5-HT in serum was not significantly affected by the level of dietary tryptophan. It suggested that the evaluation of the rate of 5-HT synthesis in fish might still rely on measuring 5-HT, 5-HIAA, and the ratio of 5-HIAA/5-HT in brain. Since 5-HT can be degraded to 5-HIAA by monoamine oxidase (MAO) [53], its activity and gene expression was further analyzed in this study. MAO activity in serum as well as its gene expression in brain increased at first and thereafter showed a decreasing trend with the increase of dietary tryptophan, while its gene expression in intestine was downregulated when an excess of tryptophan was added to the diets. It indicated that excessive tryptophan in feed, especially when reaching 23.70 g kg^−1^ of dry diet, might result in a negative feedback response in pufferfish, thereby downregulating MAO and affecting the level of 5-HT. Taken together, the regulation of 5-HT metabolism by an excess of tryptophan in feed may be an important factor mitigating the cannibalism of pufferfish [20].

In conclusion, when the level of dietary tryptophan exceeded the requirement of pufferfish and reached a certain amount (23.7 g kg^−1^ tryptophan of dry diet, corresponding to corresponding to 50.3 g kg^−1^ of dietary protein), intraspecific cannibalism would be mitigated and fish growth would be enhanced. And, the effect of tryptophan on cannibalistic behavior of pufferfish may be related to the influence of the metabolism of 5-HT. Moreover, excess tryptophan in feed caused competitive interactions with certain LNAAs such as phenylalanine, tyrosine, and methionine, thereby affecting the transport and absorption of these amino acids in pufferfish.

## Figures and Tables

**Table 1:** Formulation and proximate composition of the experimental diets (g kg^−1^).

Ingredients	T1	T2	T3	T4
Fish meal	150	150	150	150
Brewer's yeast	100	100	100	100
Corn protein concentrate	80	80	80	80
Gelatin	80	80	80	80
Wheat meal	340	340	340	340
Tryptophan	0	3	9	18
Glycine	12	10	7	3
Glutamic acid	11.4	10.4	7.4	2.4
Amino acid premix^a^	110.4	110.4	110.4	110.4
Mineral premix^b^	5	5	5	5
Vitamin premix^c^	10	10	10	10
Ca (H_2_PO_4_)_2_	20	20	20	20
Vitamin C	5	5	5	5
Choline chloride	2	2	2	2
DMPT^d^	3	3	3	3
Ethoxyquin	0.2	0.2	0.2	0.2
Calcium propionate	1	1	1	1
Fish oil	60	60	60	60
Soybean lecithin	10	10	10	10
Proximate composition				
Dry matter	931.7	928.9	934.8	939.3
Crude protein	476.8	473.4	478.9	470.8
Crude lipid	78.6	79	77.5	76.9
Ash	61.3	60.8	61.1	59.8

^a^Amino acid premix (g kg^−1^ dry diet; all L-form amino acids unless otherwise indicated): taurine, 5.0 g; aspartic acid, 10.0 g; threonine, 2.9 g; serine, 10.0 g; alanine, 10.0 g; valine, 7.3 g; D/L-Methionine, 5.0 g; isoleucine, 8.2 g; tyrosine, 11.3 g; phenylalanine, 1.9 g; lysine 21.6 g; histidine, 3.0 g; arginine, 7.7 g; leucine 6.5. Crystalline amino acids were purchased from Hebei Huayang Amino Acids Group Company Limited. ^b^Vitamin premix (IU or g/kg premix): Vitamin A acetate, 1,140,000 IU; Vitamin D3, 180,000 IU; DL-*α*-tocopherol acetate, 7.6 g; menadione, 1.2 g; thiamin nitrate, 0.93 g; riboflavin, 1.35 g; pyridoxine hydrochloride, 1.10 g; cyanocobalamine, 0.0075 g; D-calcium pantothenate, 4.5 g; nicotinamide, 6.75 g; folic acid, 0.465 g; D-biotin, 0.0475 g; inositol, 10 g. ^c^Mineral premix (g kg^−1^ premix): FeSO_4_·H_2_O, 112.7 g; ZnSO_4_·H_2_O, 45.2 g; MnSO_4_·H_2_O, 9.3 g; CuSO_4_·5H_2_O, 3.7 g; CoCl_2_·6H_2_O, 0.4 g; Na_2_SeO_3_, 0.1 g; Ca(IO_3_)_2_, 0.3 g. ^d^DMPT: dimethyl-*β*-propiothetin.

**Table 2:** Amino acid composition of the experiment diets (g kg^−1^ dry diet).

	T1	T2	T3	T4
Essential amino acid				
Threonine	13.58	13.53	13.93	12.96
Valine	20.15	20.05	20.34	19.41
Methionine	11.33	10.07	12.04	11.43
Isoleucine	17.69	15.98	17.33	17.22
Leucine	28.47	27.36	28.50	28.02
Phenylalanine	15.60	16.99	16.65	16.84
Lysine	30.57	29.67	28.95	29.67
Histidine	10.50	9.60	8.33	11.71
Arginine	30.70	38.53	38.79	37.37
Tryptophan	4.30	7.80	14.90	23.70
Non-essential amino acid				
Taurine	8.34	7.90	7.59	7.50
Aspartic acid	31.42	30.90	31.13	30.01
Serine	20.15	20.19	20.29	19.70
Glutamic acid	66.78	64.46	63.27	58.31
Glycine	37.00	34.18	31.50	27.64
Alanine	29.31	28.80	28.81	27.37
Cystine	3.74	4.36	5.36	3.94
Tyrosine	19.62	18.55	19.97	20.13
Proline	12.78	12.78	13.58	14.10

**Table 3:** Primer sequences used for qRT–PCR.

Gene	Primer sequence (5′–3′)	Product size (bp)	Accession number^1^
TPH1	F: CTCGGTGCAGAAACTGGCTA	281	NM_001032676.1
	R: AGGGACGCTTGATGGTCTTG		
DDC	F: TTACGTCACATCCAGCGAGC	200	XM_003965983.3
	R: AGTGGACAGATGCTGCCAATTT		
MAOA	F: GTCCACGGAAAGTCCTACCC	107	XM_003961571.3
	R: CTGCCCATCTTGTCTAGCGT		
TAT1	F: GGAGCTGCAAGGAGATCAAAC	290	XM_029849142.1
	R: AAACACCAAGCAGGGACACT		
B0AT1	F: CAGGACTGGAGGAACGGATT	94	XM_003969025.3
	R: TGGGCTTTGTTGTCCCACTT		
B0AT2	F: TACTCCGCTACACTGCCTCT	162	XM_003967765.3
	R: CAGGTTGGGCGTGATGTACT		
4F2hc	F: GAAATGGACCCCGAGAAGCA	173	XM_011606741.2
	R: AAACGGGTTCTCACCCATCC		
LAT2	F: TGGGGGTCTACTGGGACAAT	180	XM_003978564.3
	R: ATTTGTGGTTCTCTTCATC		
y^+^LAT2	F: CCTCGCTTTCATCCGTCTGT	292	XM_029840538.1
	R: CGTGTAGCCTTGTCCGATCT		
RPSD	F: GAAACGCTGCTCAACTCGGA	157	XM_003968784.3
	R: CAGCGATGGTCTCTCGGTTC		
*β*-actin	F: ACACTGTGCTGTCTGGAGGT	127	XM_003964421.3
	R: GAGTATTTACGCTCAGGTGGGG		

*Note*. TPH1 = tryptophan hydroxylase 1; DDC = dopa decarboxylase; MAOA = monoamine oxidase; TAT1 = T-type amino acid transporter (SLC16A10); B0AT1 = B0 neutral amino acid transporter 1 (SLC6A19); B0AT2 = SLC6A15; 4F2hc = 4F2 cell-surface antigen heavy chain (SLC3A2); LAT2 = large neutral amino acids transporter small subunit 2 (SLC7A8); y^+^LAT2 = y^+^L-type amino acid transporter 2 (SLC7A6); RPSD = RNA polymerase II subunit D. ^1^NCBI GenBank accession no.

**Table 4:** Growth performance of obscure pufferfish fed experimental diets (mean ± SEM, *n* = 3)^a^.

	T1	T2	T3	T4
Initial body weight (g)	38.00 ± 0.23	37.18 ± 0.46	38.24 ± 0.16	37.44 ± 0.25
Final body weight (g)	62.22 ± 3.11^a^	53.95 ± 0.20^b^	56.85 ± 1.27^a,b^	61.21 ± 0.90^a^
Weight gain (%)^b^	63.64 ± 7.21^a^	45.13 ± 1.37^b^	48.65 ± 2.85^b^	63.53 ± 3.28^a^
Specific growth rate (% d^−1^)^c^	0.78 ± 0.07^a^	0.59 ± 0.01^b^	0.63 ± 0.03^b^	0.78 ± 0.03^a^
Feed intake (% d^−1^)^d^	1.83 ± 0.06	1.70 ± 0.12	1.46 ± 0.12	1.83 ± 0.13
Feed efficiency ratio^e^	0.42 ± 0.05	0.35 ± 0.02	0.43 ± 0.05	0.42 ± 0.02
Protein efficiency ratio (%)^f^	1.08 ± 0.88	0.69 ± 0.73	0.76 ± 0.9	0.87 ± 0.89
Protein productive value (%)^g^	14.73 ± 2.18	14.24 ± 0.61	14.41 ± 1.69	15.10 ± 0.62
Survival rate (%)^h^	67.50 ± 1.44	65.83 ± 0.83	68.33 ± 0.83	68.33 ± 3.00
The rate of damage (%)^i^	45.72 ± 2.70^a,b^	50.71 ± 3.84^a^	47.53 ± 1.63^a,b^	36.50 ± 2.36^b^

^a^Values in the same row followed by different superscript letters are significantly different (*P* < 0.05).

**Table 5:** Whole-body proximate composition and physical indicators of obscure pufferfish^a^.

	T1	T2	T3	T4
Whole-body proximate composition				
Moisture (%)	69.65 ± 0.74	69.61 ± 0.23	70.69 ± 0.63	70.70 ± 0.55
Crude protein (%)	16.73 ± 0.21^a,b^	17.61 ± 0.29^a^	16.48 ± 0.05^b^	16.80 ± 0.20^a,b^
Crude lipid (%)	10.41 ± 0.59	10.69 ± 0.31	10.32 ± 0.51	9.79 ± 0.42
Ash (%)	2.67 ± 0.08^a,b^	2.81 ± 0.04^a^	2.56 ± 0.06^b^	2.75 ± 0.01^a,b^
Physical indicators				
Hepatosomatic index (%)^b^	15.22 ± 0.25	15.03 ± 0.4	15.22 ± 0.28	14.28 ± 0.44
Viscerasomatic index (%)^c^	20.02 ± 0.62	18.22 ± 0.88	17.8 ± 0.87	18.05 ± 0.26
Condition factor^d^	4.17 ± 0.08	3.96 ± 0.17	3.97 ± 0.06	3.89 ± 0.28

^a^Values are present as mean values ± standard error of mean (SEM) (*n* = 3). Values in the same row followed by different superscript letters are significantly different (*P* < 0.05). ^b^Hepatosomatic index = 100 × wet liver weight/fish body weight. ^c^Viscerasomatic index = 100 × wet viscera weight/fish body weight. ^d^Condition factor = 100 × body weight/(total body length).

**Table 6:** Free amino acid concentrations in brain (*μ*g g^−1^)^a^.

	T1	T2	T3	T4
Essential amino acid				
Threonine	136.79 ± 20.06	149.37 ± 27.6	117.12 ± 18.61	99.22 ± 15.48
Valine	47.6 ± 1.58	48.67 ± 1.95	45.58 ± 0.61	45.49 ± 1.00
Methionine	6.28 ± 1.24^a,b^	10.23 ± 0.91^a^	3.45 ± 0.47^b^	5.84 ± 1.76^a,b^
Isoleucine	25.48 ± 0.74	24.89 ± 2.85	22.74 ± 2.02	21.44 ± 1.37
Leucine	48.55 ± 0.39	43.96 ± 4.66	43.77 ± 2.46	39.7 ± 2.92
Phenylalanine	131.71 ± 5.88^a,b^	143.85 ± 6.96^a^	111.48 ± 4.54^b^	128.07 ± 6.39^a,b^
Lysine	60.36 ± 4.01	66.68 ± 8.95	76.61 ± 10.55	58.92 ± 2.58
Histidine	303.54 ± 29.21^b^	321.50 ± 51.70^b^	444.75 ± 13.45^a,b^	616.39 ± 45.98^a^
Arginine	64.64 ± 2.64	74.02 ± 2.91	67.03 ± 2.06	68.04 ± 0.57
Tryptophan	12.94 ± 0.53^a,b^	11.58 ± 0.77^b^	15.04 ± 0.48^a^	12.21 ± 0.52^b^
Nonessential amino acid				
Taurine	6318.05 ± 675.73	4963.28 ± 647.21	4935.22 ± 636.86	4523.96 ± 953.12
Aspartic acid	273.77 ± 37.27	234.85 ± 13.18	223.73 ± 28.04	220.86 ± 24.38
Serine	83.05 ± 7.82	97.14 ± 16.22	92.53 ± 2.10	78.29 ± 15.53
Glutamic acid	1648.37 ± 230.99	1417.55 ± 134.72	1530.01 ± 146.28	1422.09 ± 132.15
Glycine	117.14 ± 17.41	113.72 ± 9.62	108.96 ± 6.25	100.02 ± 17.21
Alanine	133.99 ± 2.96	133.36 ± 21.3	128.6 ± 8.92	133.47 ± 15.74
Cystine	17.95 ± 1.20^c^	32.49 ± 1.59^a^	25.06 ± 0.50^b^	27.11 ± 1.03^b^
Tyrosine	39.06 ± 2.14^a,b^	50.40 ± 3.89^a^	30.64 ± 1.70^b^	38.33 ± 1.71^b^
Proline	1487.53 ± 210.16	1274.78 ± 124.93	1384.65 ± 134.48	1285.2 ± 108.98

^a^Values are present as mean values ± standard error of mean (SEM) (*n* = 3). Values in the same row followed by different superscript letters are significantly different (*P* < 0.05).

**Table 7:** The concentration of 5-hydroxytryptamine and 5-hydroxyindole acetic acid in serum^a^.

	T1	T2	T3	T4
5-HT (pg ml^−1^)	98.14 ± 4.83	95.14 ± 5.60	90.53 ± 5.16	93.45 ± 7.51
5-HIAA (ng L^−1^)	48.64 ± 1.00	43.42 ± 2.82	44.43 ± 3.59	43.19 ± 1.60
5-HIAA/5-HT	497.07 ± 15.14	456.61 ± 14.97	494.05 ± 47.92	465.56 ± 20.89

*Note*. 5-HT = 5-hydroxytryptamine; 5-HIAA = 5-hydroxyindole acetic acid. ^a^Values are present as mean values ± standard error of mean (SEM) (*n* = 3). Values in the same row followed by different superscript letters are significantly different (*P* < 0.05).

## Data Availability

The data are available upon reasonable request to the corresponding author.
